# Association between serum PLP levels and the natural resolution of nausea and vomiting in pregnancy: a secondary analysis

**DOI:** 10.3389/fnut.2026.1745093

**Published:** 2026-02-09

**Authors:** Zhuwei Gao, Hong Yu, Jiannan Yu, Jing Cong, Jiaxing Feng, Yu Yao, Zihan Li, Baichao Shi, Fengjuan Lu, Xiaoke Wu, Jingshu Gao

**Affiliations:** 1 Graduate School, Heilongjiang University of Chinese Medicine, Harbin, Heilongjiang, China; 2 Zhejiang Provincial Hospital of Chinese Medicine and Zhejiang Chinese Medical University, Zhejiang, Hangzhou, China; 3 Department of Gynecology, First Affiliated Hospital, Heilongjiang University of Chinese Medicine, Harbin, Heilongjiang, China

**Keywords:** dose–response relationship, nausea and vomiting in pregnancy, pyridoxal 5′-phosphate (PLP), survival analysis, vitamin B6

## Abstract

**Background:**

Nausea and vomiting in pregnancy (NVP) are common clinical symptoms in early gestation. Although vitamin B6 supplementation has been shown to alleviate NVP, the physiological significance of its active form, pyridoxal 5′-phosphate (PLP), and its association with the natural course of symptom resolution remain unclear.

**Objective:**

To investigate the association between serum PLP levels in early pregnancy and the natural resolution of NVP, and to elucidate its potential physiological mechanisms to support individualized intervention strategies.

**Methods:**

352 pregnant women with NVP symptoms were enrolled at ≤12 gestational weeks. The exposure variable was serum PLP concentration, categorized into quartiles (Q1–Q4). The primary outcome was the time to spontaneous NVP resolution, defined by a “7-day confirmation criterion.” Cumulative remission rates were compared across quartiles using Kaplan–Meier analysis. Multivariate Cox proportional hazards models were constructed with stratified control for treatment arms (A–D), and restricted cubic spline (RCS) functions were applied to examine potential dose–response relationships.

**Results:**

Kaplan–Meier analysis revealed significant differences in cumulative remission rates among PLP quartiles (log-rank *p* = 0.00082). Compared with Q1, participants in Q4 showed a 46% lower risk of persistent symptoms (HR = 0.54, 95% CI: 0.38–0.77, *p* < 0.001). In Model 3, the protective association remained independent and significant (HR = 0.60, 95% CI: 0.42–0.86, *p* = 0.006). RCS analysis indicated a linear dose–response relationship between PLP and time to remission (*P*overall < 0.01, *P*nonlinear > 0.4).

**Conclusion:**

Higher serum PLP levels in early pregnancy were significantly associated with a faster rate of spontaneous remission of NVP. The observed linear association suggests that the active form of vitamin B6 may regulate NVP progression, reflecting the biochemical basis of natural symptom resolution. PLP may be a potential biomarker for evaluating NVP severity and predicting individualized therapeutic response.

## 1 Introduction

NVP comprises common symptoms that typically begin between 6 and 8 weeks of gestation and gradually subside by 16–20 weeks (1). Characterized by persistent vomiting accompanied by dehydration, electrolyte imbalance, ketosis, nutritional deficiency, and weight loss, NVP can become severe enough to require prolonged hospitalisation and enteral or parenteral nutritional support. These symptoms affect women’s physical health and ability to perform daily and occupational activities and disrupt interactions with their children, family members, and peers (2). Reported incidence rates range from 50 to 80% for nausea, and 50% for vomiting or retching, and a recent meta-analysis confirmed an overall prevalence of approximately 70% (3).

Currently, treatment strategies for NVP depend on symptom severity: mild cases are managed with ginger, P6 acupressure, or vitamin B6; moderate cases with vitamin B6–doxylamine combinations, antihistamines, or dopamine receptor antagonists; and severe cases or hyperemesis gravidarum (HG) require ondansetron, corticosteroids, and intravenous rehydration (2). Vitamin B6 was first introduced for the management of NVP in the 1940s, and Bendectin®, a classic combination therapy containing doxylamine and pyridoxine (vitamin B6), remains the only FDA-approved medication for the treatment of nausea and vomiting in pregnancy (4). Vitamin B6 comprises three phosphorylated pyridine derivatives—PLP, pyridoxamine 5′-phosphate, and others—of which PLP is the biologically active form. As a cofactor for more than 140 enzymes, PLP is involved in immune regulation, neurotransmission, and lipid and amino acid metabolism, and it is recognized as the most sensitive biomarker of vitamin B6 status in humans (5). In recent years, multiple trials have confirmed the clinical efficacy of vitamin B6 (pyridoxine) in relieving NVP symptoms (6, 7).

However, research on the relationship between serum vitamin B6 levels and NVP remains limited. Although numerous clinical trials have demonstrated that exogenous vitamin B6 supplementation effectively alleviates NVP symptoms, most studies have focused on the interventional rather than the physiological aspect. Few have examined endogenous vitamin B6 levels and their clinical significance, particularly its active coenzyme form, PLP. To date, no systematic study has evaluated the association between serum vitamin B6 concentrations and the severity and spontaneous resolution of NVP. The present study is based on longitudinal data from a multicenter randomized controlled trial (RCT) and focuses on the biological regulation underlying symptom persistence and natural resolution. Specifically, we analyze the association between serum PLP levels and the timing of spontaneous symptom resolution. By linking endogenous biochemical status to disease trajectory rather than solely to therapeutic response, this study aims to elucidate the heterogeneity of NVP and its potential biological mechanisms, thereby providing new insights for future translational research.

## 2 Methods and materials

This study is a secondary analysis of data derived from the NVPAct multicenter RCT. Reproducibility is ensured through transparent reporting of the trial design, study population, outcome definitions, and statistical analysis methods.

### 2.1 Participants

This study was a secondary retrospective analysis of data from the multicenter, prospective clinical trial NVPAct (NCT04401384) conducted in China. NVPAct was a large-scale, multicenter, double-blind, RCT carried out between 2020 and 2022 across 13 participating centers in mainland China. Participants were randomly assigned to four groups (Groups A–D), with detailed grouping schemes described in the original trial publication. A total of 533 pregnant women were screened for eligibility, of whom 352 were ultimately included in the present analysis (8).

### 2.2 Inclusion and exclusion criteria

The original trial enrolled outpatients presenting with NVP during early gestation (≤12 weeks). Women with severe comorbidities, a history of malignancy, or primary psychiatric disorders were excluded. Detailed study design and inclusion/exclusion criteria are described elsewhere (8).

### 2.3 Key variables

#### 2.3.1 Exposure variable

The primary exposure was serum PLP (nmol/L). Two analytic approaches were used: (1) as a continuous variable, using raw measured values to assess dose–response relationships between PLP levels and outcomes; and (2) as a categorical variable, divided into quartiles (Q1–Q4) based on the sample distribution, with Q1 (lowest quartile) as the reference group. We categorized baseline serum PLP into quartiles because no clearly defined clinical PLP cut-off is currently available for predicting the course of NVP. Quartile-based categorization avoids arbitrary threshold selection and ensures balanced group sizes, thereby enabling more stable estimation and interpretation of the results.

#### 2.3.2 Outcome variable

The primary outcome was the time to NVP remission. Remission was defined using a prespecified “7-day confirmation criterion”: an event (remission) was recorded when the participant first achieved a Pregnancy-Unique Quantification of Emesis (PUQE) score ≤6 (According to the validated PUQE scoring system, a total score ≤6 corresponds to mild NVP, which is generally considered clinically tolerable and has been widely used as a threshold to define symptom remission in both observational and interventional studies.) (9). At least one subsequent assessment within the next seven consecutive calendar days (including day 7) also remained ≤6. The date of the first achievement was defined as the event date. This criterion was established to avoid transient fluctuations and ensure clinical stability of remission. Participants who did not meet this criterion during follow-up were right-censored at the last contact date, defined as the latest recorded PUQE assessment or the date of withdrawal, loss to follow-up, or adverse pregnancy event—whichever occurred last. The starting point (t0) was the informed consent/enrollment date, and time was measured in days.

#### 2.3.3 Covariates and stratification factors

Covariates were selected based on a directed acyclic graph (DAG, Supplementary Figure S1) and prior evidence to construct the minimal sufficient adjustment set, including variables associated with NVP outcomes and potentially correlated with PLP levels. These covariates were categorised into three domains: (1) Demographic and obstetric factors: maternal age, gestational age, body mass index (BMI), parity, and plurality (singleton/twin pregnancy); (2) Symptom and psychological scales: PUQE score, Self-Rating Anxiety Scale (SAS; standard score range = 25–100, anxiety defined as ≥ 50), and Self-Rating Depression Scale (SDS; standard score range = 25–100, depression defined as ≥ 53); (3) Biochemical indicators: serum electrolytes including sodium (Na), potassium (K), and calcium (Ca) (all in mmol/L), and the neurotransmitter serotonin (5-HT, ng/mL).

Evidence supporting the associations of each covariate with NVP and/or PLP is provided in Supplementary Table S1. In addition, given that the present study is a secondary analysis of an RCT, the four original treatment groups (Groups A–D) may have influenced remission of NVP and circulating PLP levels through intervention-related mechanisms. Therefore, the treatment group was incorporated as a stratification factor in all models to control for potential confounding from the intervention and to ensure robust estimation of the association between serum PLP levels and NVP outcomes.

### 2.4 Statistical analysis

All statistical analyses were performed using IBM SPSS Statistics version 25.0 (IBM Corp., New York, USA) and R software version 4.3.1 (R Foundation for Statistical Computing, Vienna, Austria) on the RStudio platform. Missing values in biochemical variables were handled using multiple imputation with predictive mean matching. Five imputed datasets were generated, and the completed dataset was used for subsequent analyses. Outcome variables and follow-up time were not imputed. The normality of continuous variables was assessed using the Shapiro–Wilk test. Normally distributed variables were presented as mean ± standard deviation (SD) and compared between groups using the independent-samples t-test; the Welch correction was applied when homogeneity of variance was violated, and mean differences with 95% confidence intervals (CIs) were reported. Non-normally distributed variables were expressed as median (interquartile range, IQR) and compared using the Mann–Whitney U test, with median differences and 95% CIs estimated via bootstrapping (2,000 resamples). Categorical variables were presented as frequencies (percentages) and compared using the chi-square test or Fisher’s exact test, as appropriate.

The outcome event was the time to NVP remission. Kaplan–Meier methods were used to plot survival curves, and group differences were assessed with the log-rank test. Multivariate Cox proportional hazards models were sequentially constructed under stratified control for treatment arms (Groups A–D): Model 1 included PLP only; Model 2 additionally adjusted for age, gestational age, BMI, parity, and plurality; and Model 3 further adjusted for PUQE score, SAS, SDS, Na, K, Ca, and 5-HT. In the Cox regression models, the event was defined as persistent NVP symptoms (i.e., failure to achieve remission). Hence, a hazard ratio below 1 indicates a lower risk of symptom persistence and a faster remission. Results were expressed as hazard ratios (HRs) with 95% CIs, and proportional hazard assumptions were tested using Schoenfeld residuals.

To explore potential dose–response relationships, RCS functions were applied to assess both linear and nonlinear effects of PLP on remission risk, with overall and nonlinearity *p* values reported. Prespecified subgroup analyses were also performed under Model 3, stratified by demographic, obstetric, and psychological factors, and visualized as forest plots to evaluate interaction effects. All statistical tests were two-sided, and *p* < 0.05 was considered statistically significant.

## 3 Results

### 3.1 Baseline characteristics across quartiles of PLP

A total of 352 participants were included in this study. Based on baseline serum PLP levels, participants were categorised into four quartiles: Q1 (≤6.30 nmol/L), Q2 (6.31–9.10 nmol/L), Q3 (9.11–15.41 nmol/L), and Q4 (≥15.42 nmol/L), with 88 participants in each group. The median (interquartile range) PLP concentration for the overall population was 9.11 (6.30, 15.42) nmol/L. No significant differences were observed among the four groups in terms of age, BMI, occupational distribution, gestational age, parity, psychological scores (SAS and SDS), potassium (K), sodium (Na), and 5-HT levels (*p* > 0.05). However, significant differences were observed in PUQE scores and serum Ca levels (*p* = 0.010 and *p* = 0.020, respectively), suggesting that NVP severity and serum Ca levels varied significantly across quartile groups (Table 1).

**Table 1:** Baseline characteristics of patients across quartiles of PLP.

Variable (unit, summary type)	Q1 (Low)	Q2	Q3	Q4 (High)	*p*
No. of participants (*n*)	88	88	88	88	
Age (years), median (IQR)	28.73 (26, 31.75)	29.27 (25.25, 32)	28.92 (26, 32)	29.35 (26.25, 32)	0.660
BMI (kg/m^2^), median (IQR)	21.62 (19.23, 23.26)	21.52 (19.05, 23.34)	20.97 (18.68, 23.07)	21.49 (19.39, 23.16)	0.532
Occupation, *n* (%)					0.819
Employee	39 (44.3%)	36 (40.9%)	29 (33%)	31 (35.2%)	
Housewife	17 (19.3%)	17 (19.3%)	17 (19.3%)	14 (15.9%)	
Business	1 (1.1%)	3 (3.4%)	1 (1.1%)	3 (3.4%)	
Farmer	0 (0%)	1 (1.1%)	1 (1.1%)	0 (0%)	
Civil servant	3 (3.4%)	2 (2.3%)	4 (4.5%)	6 (6.8%)	
Others	28 (31.8%)	29 (33%)	36 (40.9%)	34 (38.65%)	
Gestational age (weeks), median (IQR)	8.78 (7, 10.75)	8.65 (7, 10)	8.75 (7, 10)	8.88 (7, 10)	0.761
Plurality, *n* (%)					0.489
Singleton	77 (92%)	84 (96.6%)	76 (92.7%)	83 (96.5%)	
Twin	6 (7.2%)	3 (3.4%)	6 (7.3%)	3 (3.5%)	
Parity, median (IQR)	1.30 (0, 2)	1.17 (0, 2)	1.20 (0, 2)	1.48 (0, 2)	0.598
PUQE score, mean ± SD	10.91 ± 2.25	10.52 ± 2.18	11.35 ± 2.22	11.56 ± 2.25	0.010*
SAS score, median (IQR)	42.78 (36, 49)	42.92 (37, 49)	43.72 (37.25, 48.75)	45.80 (39.25, 45)	0.134
SDS score, median (IQR)	47.08 (41, 52.75)	45.92 (40.25, 52)	47.42 (42, 53)	48.80 (44, 55)	0.230
Na (mmol/L, mean ± SD)	136.59 ± 3.24	137.08 ± 3.08	136.15 ± 3.38	136.94 ± 2.95	0.208
K (mmol/L), median (IQR)	4.05 (3.77, 4.21)	4.08 (3.81, 4.19)	4.03 (3.75, 4.27)	4.03 (3.79, 4.16)	0.622
Ca (mmol/L), median (IQR)	2.24 (2.19, 2.31)	2.28 (2.20, 2.34)	2.29 (2.24, 2.38)	2.26 (2.19, 2.36)	0.020*
5-HT (pg/mL, mean ± SD)	357.61 ± 583.76	558.95 ± 1130.35	447.06 ± 1068.85	394.20 ± 696.17	0.476

Variables with a normal distribution are presented as mean ± standard deviation (mean ± SD); variables with a non-normal distribution are presented as median (interquartile range, IQR); categorical variables are expressed as number and percentage, *n* (%). *p* values indicate the results of group comparisons, and * denotes statistical significance (*p* < 0.05). SAS, Self-Rating Anxiety Scale; SDS, Self-Rating Depression Scale.

### 3.2 Association between PLP levels and remission of NVP

**Figure 1:**
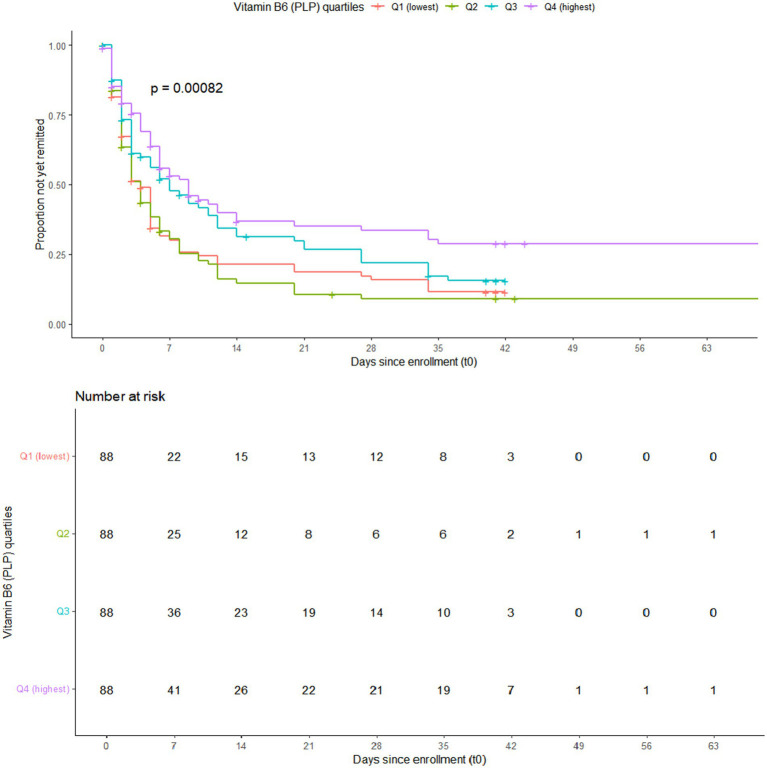
Kaplan–Meier survival curves for spontaneous remission of NVP by quartiles of PLP.

The Kaplan–Meier survival analysis revealed a significant difference in cumulative remission rates for NVP across the four PLP quartiles (log-rank *p* = 0.00082). Overall, higher PLP levels were associated with a greater probability of remission, with the highest quartile (Q4) demonstrating the earliest symptom relief (Figure 1).

We plotted Kaplan–Meier curves of natural remission stratified by treatment arms (A–D), as part of a sensitivity analysis The results showed significant differences in remission rates across treatment arms when PLP was not included in the model (log-rank *p* = 0.0062), suggesting that the type of intervention might influence the course of symptom relief (Supplementary Figure S2). Therefore, to further validate the robustness of this association, we incorporated treatment groups and other covariates into multivariable Cox regression models to adjust for them. Based on Schoenfeld residuals, no evidence of violation of the proportional hazards assumption was observed in the fully adjusted Cox regression model (global test *p* = 0.49).

### 3.3 Association between PLP levels and time to remission

The treatment group was included as a covariate in all multivariate Cox regression models to control for potential confounding by treatment allocation on symptom remission. In the Cox regression analysis, higher PLP levels were significantly associated with earlier remission of NVP. When analysed as a continuous variable, each unit increase in PLP was associated with a reduced hazard of persistent symptoms across all models (HR = 0.98, 95% CI: 0.97–0.99, *p* < 0.01). When categorised into quartiles, the highest quartile (Q4) consistently showed a markedly lower risk of delayed remission than the lowest quartile (Q1). Specifically, in Model 1, Q4 vs. Q1 yielded HR = 0.54 (95% CI: 0.38–0.77, *p* < 0.001), in Model 2 HR = 0.54 (95% CI: 0.38–0.78, *p* < 0.001), and in Model 3 HR = 0.60 (95% CI: 0.42–0.86, *p* = 0.006). A clear linear trend was observed across quartiles (*P* for trend < 0.01 in all models) (Table 2).

**Table 2:** Association between PLP levels and time to remission in Cox regression models.

Exposure	Model 1		Model 2		Model 3	
	HR (95% CI)	*p*	HR (95% CI)	*p*	HR (95% CI)	*p*
PLP as continuous	0.98 (0.97–0.99)	<0.001*	0.98 (0.97–0.99)	<0.001*	0.98 (0.97–0.99)	0.001*
Q1 (Reference)	1.00 (Reference)		1.00 (Reference)		1.00 (Reference)	
Q2	1.05 (0.76–1.46)	0.754	1.11 (0.80–1.56)	0.529	1.04 (0.74–1.47)	0.809
Q3	0.72 (0.51–1.00)	0.053	0.76 (0.54–1.08)	0.130	0.72 (0.50–1.03)	0.071
Q4	0.54 (0.38–0.77)	<0.001*	0.54 (0.38–0.78)	<0.001*	0.60 (0.42–0.86)	0.006*
*P* for trend		<0.001*		<0.001*		0.001*

All models were stratified by treatment arms (A–D). Model 1: univariate analysis including PLP only; Model 2: adjusted for age, gestational age, BMI, parity, and plurality; Model 3: further adjusted for PUQE score, SAS, SDS, Na, K, Ca, and 5-HT. * denotes all statistically significant *p* values.

**Figure 2:**
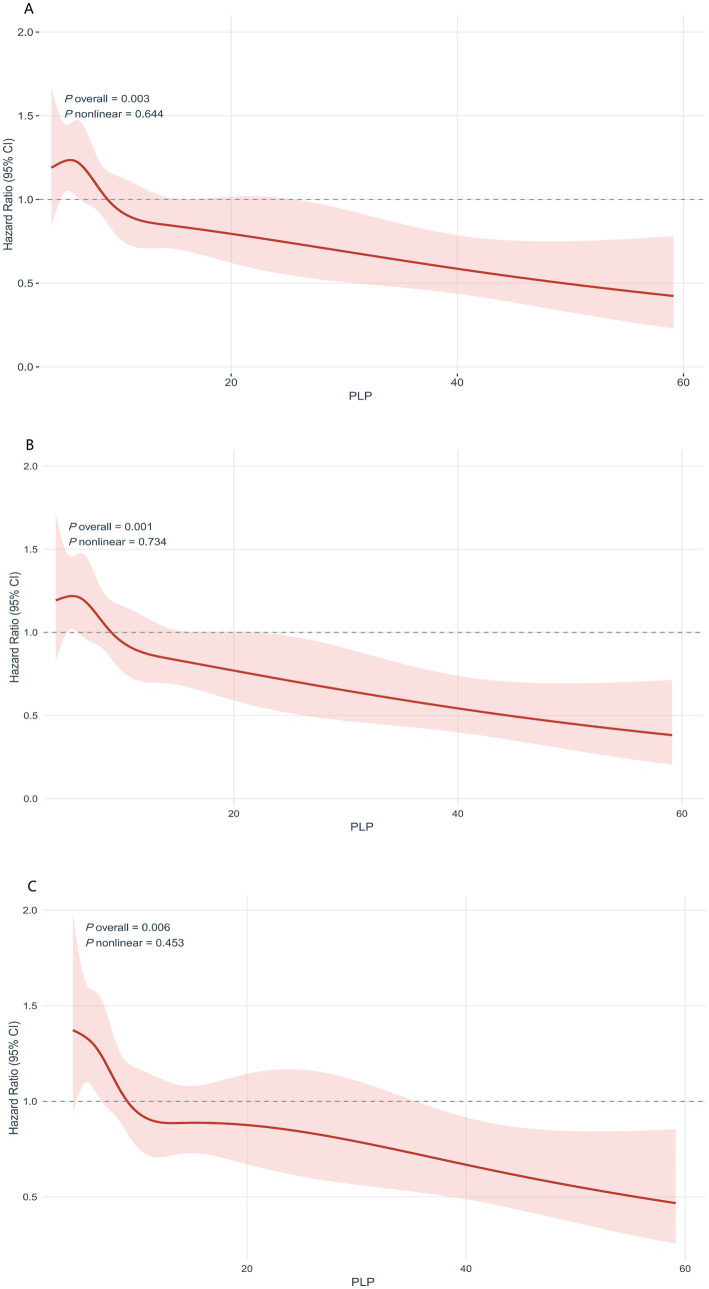
RCS curves for the association between PLP and time to remission across three Cox regression models. RCS curves illustrate the dose–response relationship between PLP (continuous variable) and remission time. **(A)** Model 1; **(B)** Model 2; **(C)** Model 3. HR was referenced to the median PLP level, with red lines indicating estimated values and pink shaded areas representing the 95% confidence intervals.

RCS analysis further illustrated this linear dose–response association. Overall, *p*-values were statistically significant across all models (*P*overall < 0.01), while no evidence of nonlinearity was detected (*P*nonlinear > 0.4). The spline curves confirmed that increasing PLP concentrations were linearly associated with a reduced hazard of persistent nausea and vomiting. Notably, the RCS curves for Models 1 and 2 overlapped almost entirely, indicating that adjustment for age, gestational age, BMI, parity, and plurality did not materially alter the linear association between PLP and the risk of symptom persistence (Figure 2).

### 3.4 Subgroup analyses based on fully adjusted model (Model 3)

As a supplementary analysis, subgroup analyses were conducted to explore the consistency of the association between baseline serum PLP and symptom persistence across clinically relevant strata. In the multivariate logistic regression analysis, age <29 years (OR = 0.97, 95% CI: 0.95–0.99, *p* < 0.001), BMI < 21.1 (OR = 0.96, 95% CI: 0.94–0.98, *p* < 0.001), gestational age ≥8 weeks (OR = 0.98, 95% CI: 0.96–0.99, *p* < 0.001), PUQE score ≥11 (OR = 0.98, 95% CI: 0.97–0.99, *p* = 0.005), and SDS score ≥53 (OR = 0.96, 95% CI: 0.94–0.99, *p* = 0.004) were significantly associated with a lower risk of symptom persistence.

**Figure 3:**
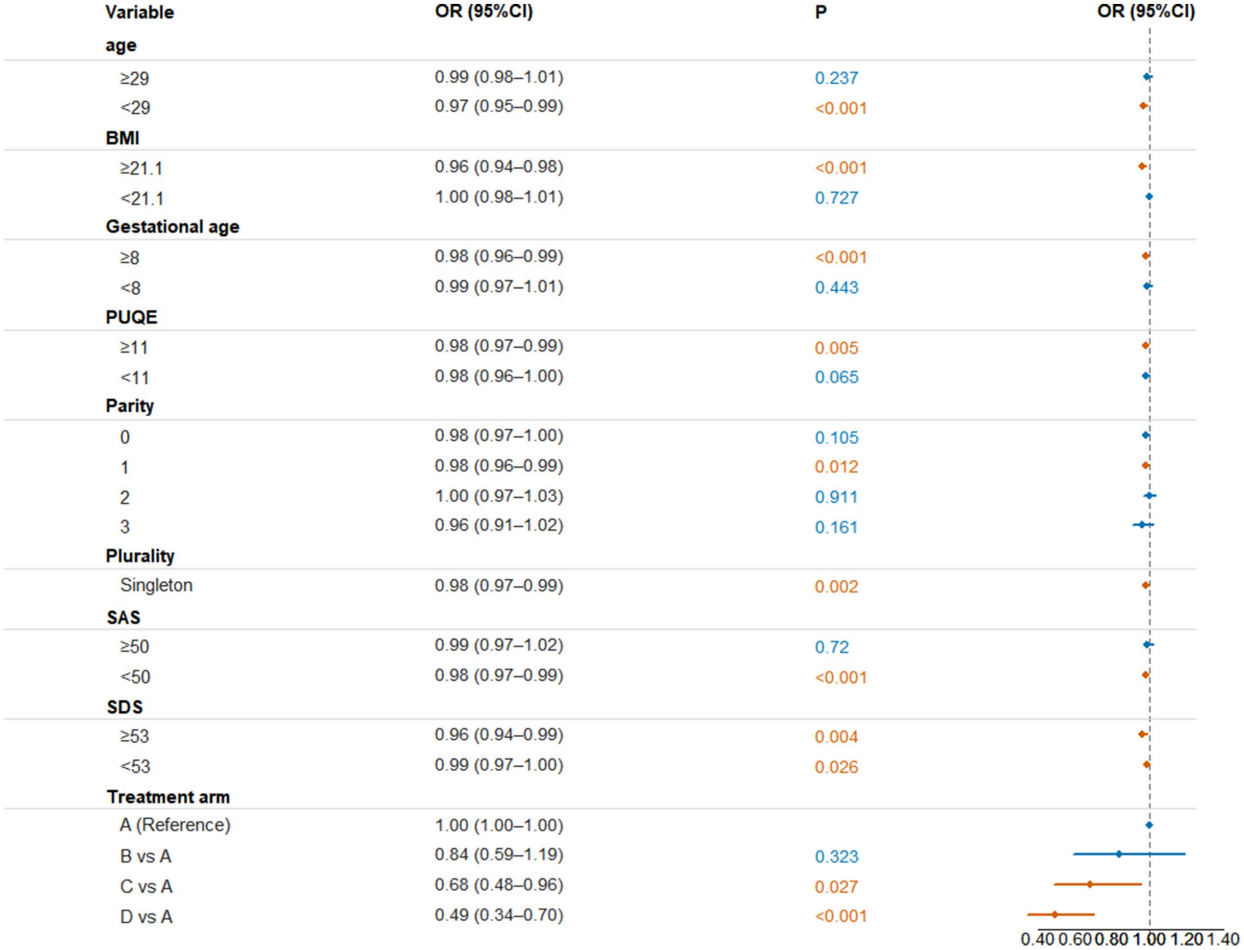
Forest plots of subgroup analyses based on the fully adjusted model (Model 3). ORs were derived from stratified logistic regression for descriptive and visualization purposes and should be interpreted alongside the primary Cox proportional hazards analyses.

Singleton pregnancies also showed a slightly reduced risk (OR = 0.98, 95% CI: 0.97–0.99, *p* = 0.002). Compared with arm A, both arm C (OR = 0.68, 95% CI: 0.48–0.96, *p* = 0.027) and arm D (OR = 0.49, 95% CI: 0.34–0.70, *p* < 0.001) showed significantly lower risks of persistent symptoms (Figure 3). Formal tests for interaction between baseline PLP and prespecified subgroup variables were conducted, and no firm evidence of effect modification was observed (Supplementary Table S3).

## 4 Discussion

This secondary analysis of the multicentre randomized NVPAct trial found that higher serum PLP levels in early pregnancy were associated with faster spontaneous remission of NVP. Survival and multivariate Cox analyses confirmed the independent protective effect of PLP, and restricted cubic spline modeling revealed a linear dose–response relationship, suggesting that PLP reflects the biochemical regulation underlying natural recovery from NVP.

In recent years, multiple studies have demonstrated that vitamin B6 (pyridoxine) and its active form, PLP, exert therapeutic effects in NVP. Early RCT showed that oral pyridoxine significantly reduced nausea severity and vomiting frequency in pregnant women, supporting its short-term efficacy in moderate-to-severe NVP (10). Subsequent studies confirmed the effectiveness of pyridoxine monotherapy, reporting symptom improvement comparable to that with ginger and favorable tolerability (11). More recent evidence further supported its clinical benefit, demonstrating improved symptom severity and remission outcomes without increased risk of fetal malformations or miscarriage (12). However, most previous studies have primarily focused on the short-term efficacy of exogenous supplementation, and few have evaluated how endogenous serum PLP concentrations affect the natural course and spontaneous remission of NVP. A literature review identified only one study by Noroyono Wibowo (13), which examined plasma vitamin B6 concentrations in pregnant women with NVP and similarly found that lower plasma vitamin B6 levels were associated with more severe symptoms. However, that study did not distinguish between the active forms of vitamin B6 and used an exogenous supplementation design. Building on this foundation, the present study focused specifically on PLP—the biologically active metabolite of vitamin B6—and revealed a precise dose–response relationship and potential mechanism linking serum PLP levels to the natural remission of NVP, achieving a crucial shift from “clinical response observation” to “physiological mechanism interpretation.” Survival analyses demonstrated significant differences in cumulative remission rates across PLP quartiles (log-rank *p* = 0.00082). Compared with the lowest quartile (Q1), women in the highest quartile (Q4) had a 46% lower risk of persistent symptoms (HR = 0.54, 95% CI: 0.38–0.77, *p* < 0.001). A significant positive linear trend was observed (*P* for trend < 0.001), whereas RCS analysis showed no evidence of nonlinearity, suggesting a linear dose–response relationship. These findings indicate that the active form of vitamin B6 may not only underlie the pharmacological mechanism of conventional antiemetic therapy but also serve as an independent biochemical marker reflecting disease progression and spontaneous recovery in NVP.

Previous studies have shown that plasma PLP levels decline progressively during pregnancy compared with nonpregnant women, indicating depletion of the active form of vitamin B6 (14). This decline may be related to hemodilution caused by plasma volume expansion and the transplacental transfer of PLP to the fetus (15), suggesting that pregnant women have relative vitamin B6 deficiency throughout gestation (16, 17). Recent studies also demonstrated that vitamin B6 deficiency during pregnancy can lead to nausea, vomiting, or even HG (18). PLP is the biologically active form of vitamin B6 and functions as a key coenzyme in multiple metabolic and neurotransmitter-related pathways (19, 20). Plasma PLP concentration is an effective biomarker for assessing vitamin B6 status (14). Moreover, PLP is the primary active metabolite through which exogenous vitamin B6 exerts its antiemetic effect (21). In fact, PLP represents the biologically active form of vitamin B6, and its physiological and pharmacological activity—whether derived from exogenous supplementation or endogenous metabolism—ultimately depends on circulating PLP levels.

Multivariate Cox regression further confirmed that, after adjusting for age, gestational age, BMI, obstetric history, plurality, psychological indices, and electrolyte levels, the protective effect of PLP remained independently significant. These findings suggest that the observed association is not fully explained by metabolic or psychological factors alone, but may reflect involvement in neuroendocrine regulatory processes related to NVP. Although the mechanisms by which PLP may influence NVP remain incompletely understood, it is generally accepted that plasma vitamin B6 concentrations are associated with symptom severity. Previous studies have proposed that vitamin B6, acting as a cofactor in multiple enzymatic reactions, may influence the onset and resolution of NVP through its involvement in neurotransmitter synthesis and steroid hormone–related pathways. However, these proposed mechanisms are based mainly on indirect evidence and require further investigation for confirmation (13, 17).

From a clinical perspective, measurement of serum PLP levels may have potential prognostic and management value. NVP is primarily characterized by symptom persistence and impaired quality of life rather than hard clinical endpoints. In this disease context, a 40% reduction in the risk of symptom persistence represents a relatively large effect size, particularly given that the association remained robust after adjustment for demographic, obstetric, psychological, and biochemical factors, supporting its clinical relevance. Although this effect size does not imply a direct therapeutic benefit, our findings suggest that baseline serum PLP levels may help distinguish women more likely to experience prolonged symptoms from those more likely to experience earlier spontaneous remission. Such prognostic stratification may be clinically informative, providing a basis for anticipatory counseling in early pregnancy and for individualizing the intensity of follow-up and supportive management. Furthermore, given the favorable safety profile and wide availability of vitamin B6 in the context of NVP management, future studies establishing PLP thresholds or dynamic monitoring strategies may facilitate early identification of high-risk individuals and provide more refined biochemical support for individualized care. Ongoing monitoring of PLP levels may also help avoid the coexistence of insufficient and excessive supplementation, thereby providing an objective biochemical basis for more precise management of NVP; however, these potential applications require confirmation in prospective studies.

## 5 Strengths and limitations

This study has notable strengths. It is the first to systematically examine the association between endogenous PLP and the natural course of NVP, addressing gaps in prior studies focused mainly on exogenous pyridoxine. Using longitudinal time-to-event data, survival and spline analyses demonstrated a linear dose–response between PLP and remission risk. Hierarchical models with progressive adjustment for demographic, obstetric, psychological (SAS/SDS), and biochemical factors (Na, K, Ca, 5-HT) enhanced causal control and clinical interpretability. Despite the valuable findings of this study, several limitations should be acknowledged. First, the study population was derived from a RCT, and its participant characteristics and intervention context may differ from those of the general pregnant population. Therefore, the generalizability of the findings requires further validation in independent, real-world cohorts. Second, this study did not concurrently quantify several known or potential emetogenic factors, including dietary vitamin B6 intake, supplement use, and key endocrine markers such as GDF15 and hCG. As a result, residual confounding and measurement error cannot be entirely excluded. In addition, as a secondary analysis of a RCT, specific interventions in the parent study—particularly treatment regimens involving vitamin B6 supplementation—may have influenced circulating PLP levels. Although treatment allocation was included as a stratification factor in all statistical models to minimize intervention-related effects, residual confounding attributable to specific interventions cannot be entirely ruled out. Therefore, the results should be interpreted with appropriate caution. Finally, detailed information on the use of folic acid or multivitamin supplements was not systematically collected, and some supplements may contain vitamin B6, which could further affect serum PLP levels. Although baseline serum PLP was measured prior to outcome assessment in this study, future prospective investigations should more precisely document supplement composition, dosage, and timing to further validate and extend the present findings.

## 6 Conclusion

This multicenter real-world analysis demonstrated that higher maternal serum PLP levels were associated with a faster rate of spontaneous remission of NVP. The observed linear dose–response relationship suggests that PLP may serve as an important endogenous regulator of NVP progression and a potential target for prediction and intervention. These findings provide new biochemical evidence for understanding the pathophysiological basis of NVP and offer a scientific foundation for individualized clinical management and nutritional intervention strategies.

## Data Availability

The original contributions presented in the study are included in the article/Supplementary material, further inquiries can be directed to the corresponding authors.
