# Supplementary material for: Association between serum PLP levels and the natural resolution of nausea and vomiting in pregnancy: a secondary analysis
Source: Front Nutr. 2026 Feb 9;13:1745093. doi: 10.3389/fnut.2026.1745093 (PMC12926342; doi:10.3389/fnut.2026.1745093)
Supplement: Supplementary file 1 [file Table_1.DOCX]

****Figure 1. DAG illustrates variable relationships and models the construction strategy.****

**
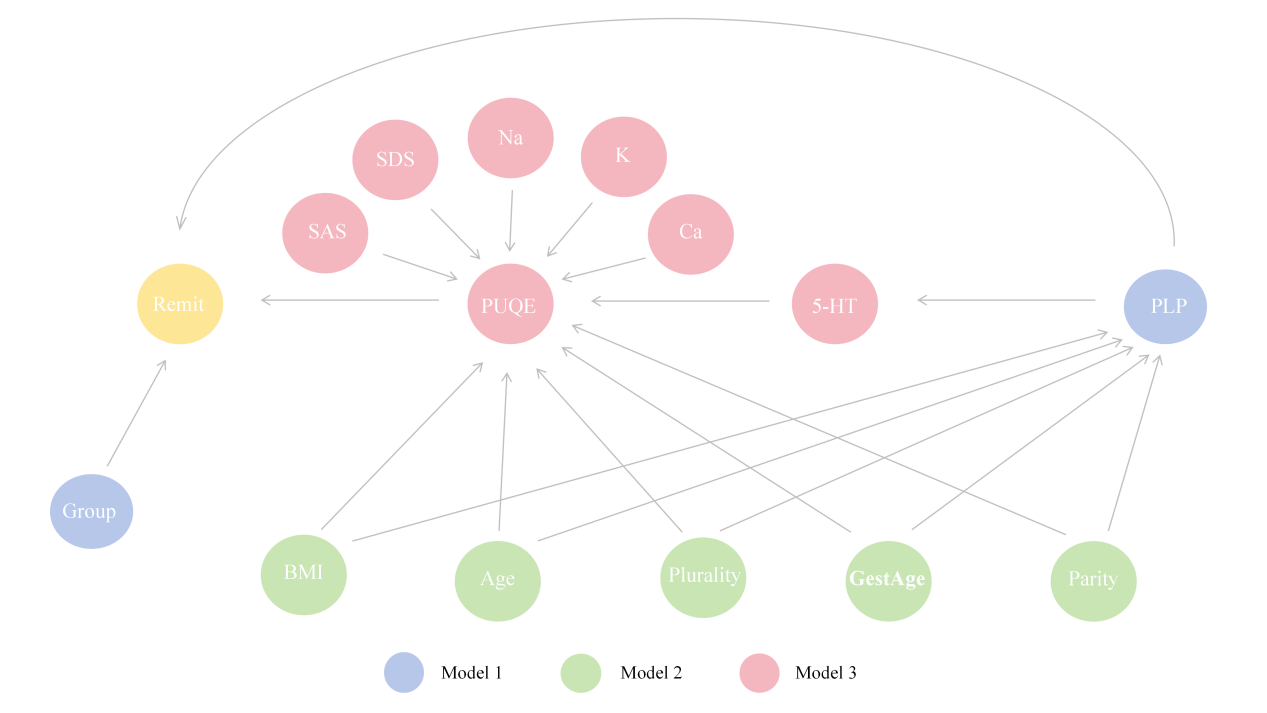
**

**Supplementary Table S1. Covariates included in the Cox regression models and supporting evidence**

| Variable | Rationale for inclusion | Evidence type | References |
| --- | --- | --- | --- |
| Age | Women younger than 25 years are more likely to experience NVP. | Secondary analysis of RCT (observational) | (1) |
|  | Younger maternal age is associated with higher risk of NVP, and with increased hospital admission and readmission for HG. | Cohort Study | (2) |
| Gestational age | Smaller gestational age is more likely to be associated with persistent moderate-to-severe NVP; as gestational age increases, the severity of NVP decreases. | Retrospective observational study | (3) |
|  | With increasing gestational age, the severity of NVP progressively decreases; moderate-to-severe NVP (including HG) predominantly occurs in early pregnancy. | cohort study | (4) |
| BMI | Higher pre-pregnancy BMI is independently associated with lower plasma vitamin B6-PLP levels. Obesity-related inflammation may accelerate PLP depletion, impairing neurotransmitter synthesis (5-HT, GABA) involved in nausea regulation. | Prospective cohort study | (5) |
|  | Higher pre-pregnancy BMI significantly increases the risk of HG as an independent predictor, while BMI during pregnancy shows no significant association. | Case–control study | (6) |
| Parity | A previous history of HG is the strongest predictor of recurrence in subsequent pregnancies, with a recurrence rate of approximately 89%. | Prospective cohort follow-up | (7) |
|  | Primiparity is associated with a higher risk of HG compared with multiparity. | cohort study | (8) |
| Plurality | Multiple pregnancy was identified as an independent risk factor for hospitalization due to HG. Compared with singleton pregnancies, the risk was significantly higher . | cohort study | (9) |
|  | Multiple pregnancy is associated with approximately double the risk of HG compared with singleton pregnancy. | cohort study | (8) |
| PUQE score | PUQE scores correlated significantly with clinical indicators of HG severity and with measures of physical and mental quality of life. Higher PUQE scores were associated with lower QoL, indicating worse symptoms. | Prospective cohort study | (10) |
|  | PUQE-24 showed excellent configural, measurement and structural invariance between nulliparas and multiparas and across two time points, strongly correlating with NVP-QOL. | Instrument validation study | (11) |
| SAS | Higher β-HCG and anxiety are significantly associated with severe HG; anxious pregnant women have ~4.9-fold higher risk of severe vomiting compared with non-anxious women. | Observational study | (12) |
| SDS | Vitamin B6 combined with acupressure significantly alleviated vomiting and reduced SAS/SDS scores, indicating that anxiety and depression play an important role in the course of NVP/HG. | RCT | (13) |
| Na | Hyponatremia frequently occurs in HG, indicating that sodium imbalance may contribute to the pathophysiology of NVP/HG. | Descriptive cross-sectional study | (14) |
|  | HG can cause severe hyponatremia, hypokalemia, and metabolic alkalosis, highlighting the importance of early recognition and fluid correction. | Case report | (15) |
| K | Potassium channels (e.g., TREK-1) participate in smooth-muscle mechanotransduction, regulating gastrointestinal motility and contractility. | Review | (16) |
|  | HG can cause severe hypokalemia leading to periodic paralysis and respiratory failure, indicating a close link between potassium imbalance and NVP severity. | Case report | (17) |
| Ca | CYP24A1 mutation–related vitamin D metabolic disorder causes pregnancy-associated hypercalcemia, presenting with nausea and vomiting. | Case report and review | (18) |
|  | Pregnancy-associated hypercalcemia may exacerbate nausea and vomiting via PTHrP-mediated calcium transport and enhanced gastrointestinal smooth-muscle excitability. | Case reports and l review | (19) |
| 5-HT | 5-HT mediates nausea–vomiting reflexes via the gut–brain axis; activation of 5-HT₃ receptors correlates with NVP/HG severity | Review | (20) |
|  | Elevated hCG increases 5-HT levels and promotes NVP; blocking 5-HT₃ receptors (e.g., ondansetron) effectively alleviates symptoms. | Review | (21) |

**Supplementary Figure 2. Survival curve of natural remission of NVP without inclusion of vitamin B6-PLP**

Supplementary analysis showed significant differences in remission rates among treatment arms when B6-PLP was not included (log-rank P = 0.0062), indicating that intervention type may influence symptom resolution. However, in multivariate Cox models adjusting for treatment group, serum B6-PLP remained an independent predictor of faster remission, suggesting that endogenous PLP level exerts a protective effect beyond exogenous supplementation.


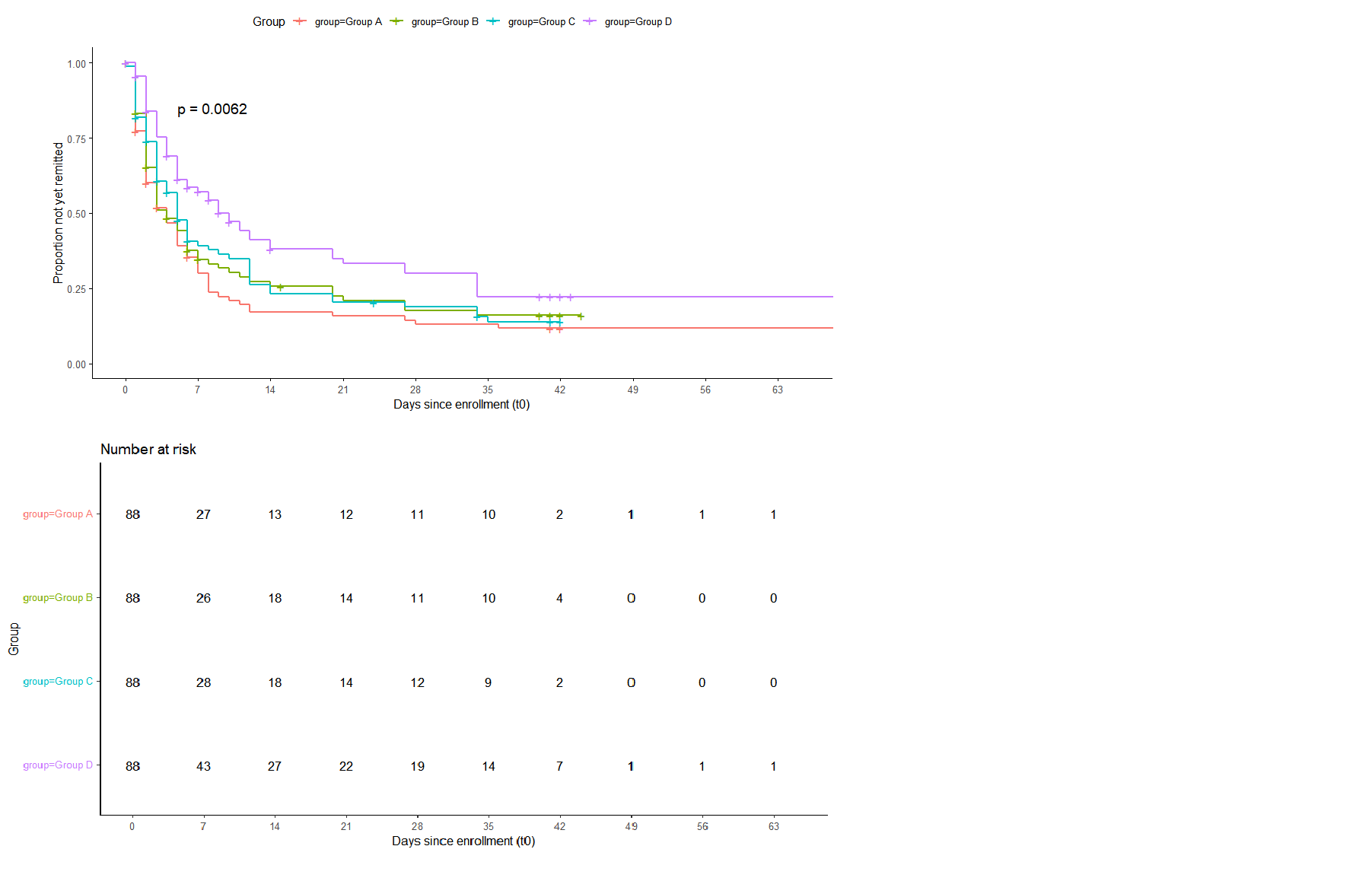


Note: The figure illustrates the cumulative probability of natural remission of NVP during follow-up. Serum vitamin B6PLP levels were not included as covariates in this model, and the curve reflects the overall natural remission trend of the study population.

**Supplementary Table S3. Formal tests for interaction between baseline serum PLP and prespecified subgroup variables in the fully adjusted Cox model**

| Subgroup variable | *P* for interaction |
| --- | --- |
| age | 0.003 |
| BMI | 0.002 |
| Gestational age | 0.269 |
| PUQE | 0.778 |
| Parity | 0.033 |
| Plurality | 0.187 |
| SAS | 0.309 |
| SDS | 0.122 |
| Treatment arm | 0.101 |

Note: *P* values for interaction were obtained from likelihood ratio tests comparing fully adjusted Cox proportional hazards models with and without interaction terms between baseline serum PLP and each subgroup variable. Interaction analyses were prespecified and conducted for exploratory purposes.

**References**

1. Hinkle SN, Mumford SL, Grantz KL, Silver RM, Mitchell EM, Sjaarda LA, et al., Association of nausea and vomiting during pregnancy with pregnancy loss: A secondary analysis of a randomized clinical trial. *JAMA internal medicine*, (2016) 176(11) 1621-1627. doi: 10.1001/jamainternmed.2016.5641

2. Fiaschi L, Nelson-Piercy C, Tata LJ, Hospital admission for hyperemesis gravidarum: A nationwide study of occurrence, reoccurrence and risk factors among 8.2 million pregnancies. *Human Reproduction*, (2016) 31(8) 1675-1684. doi: 10.1093/humrep/dew128

3. Zhang H, Wu S, Feng J, Liu Z. Risk factors of prolonged nausea and vomiting during pregnancy. *Risk Management and Healthcare Policy* (2020) 13:2645–2654. doi:10.2147/RMHP.S273791

4. Hsieh YL, Chiang CJ, Yu T. Association between nausea and vomiting during pregnancy and adverse pregnancy outcomes: findings from the nuMoM2b study. *Archives of Gynecology and Obstetrics* (2025) 312(5):1695–1704. doi:10.1007/s00404-025-08176-3

5. Bjørke-Monsen A-L, Ulvik A, Nilsen RM, Midttun Ø, Roth C, Magnus P, et al., Impact of pre-pregnancy bmi on b vitamin and inflammatory status in early pregnancy: An observational cohort study. *Nutrients*, (2016) 8(12) 776. doi:10.3390/nu8120776

6. Kosus A, Eser A, Kosus N, Usluogullari B, Hizli D, Hyperemesis gravidarum and its relation with maternal body fat composition. *Journal of Obstetrics and Gynaecology*, (2016) 36(6) 822-826. doi: 10.3109/01443615.2016.1157153

7. Nijsten K, Dean C, van der Minnen LM, Bais JM, Ris‐Stalpers C, van Eekelen R, et al., Recurrence, postponing pregnancy, and termination rates after hyperemesis gravidarum: Follow up of the mother study. *Acta Obstetricia et Gynecologica Scandinavica*, (2021) 100(9) 1636-1643. doi: 10.1111/aogs.14197

8. Pont S, Bond DM, Shand AW, Khan I, Zoega H, Nassar N, Risk factors and recurrence of hyperemesis gravidarum: A population‐based record linkage cohort study. *Acta Obstetricia et Gynecologica Scandinavica*, (2024) 103(12) 2392-2400. doi: 10.1111/aogs.14966

9. Kim HY, Cho GJ, Kim SY, Lee K-M, Ahn KH, Han SW, et al., Pre-pregnancy risk factors for severe hyperemesis gravidarum: Korean population based cohort study. *Life*, (2020) 11(1) 12. doi: 10.3390/life11010012

10. Laitinen L, Nurmi M, Kulovuori N, Koivisto M, Ojala E, Rautava P, et al., Usability of pregnancy-unique quantification of emesis questionnaire in women hospitalised for hyperemesis gravidarum: A prospective cohort study. *BMJ open*, (2022) 12(5) e058364. doi: 10.1136/bmjopen-2021-058364

11. Hada A, Minatani M, Wakamatsu M, Koren G, Kitamura T. The pregnancy-unique quantification of emesis and nausea (PUQE-24): configural, measurement, and structural invariance between nulliparas and multiparas and across two measurement time points. *Healthcare* (Basel) (2021) 9(11):1553. doi:10.3390/healthcare9111553

12. Suhaid DN, Sunjaya DK, Lubis VMT, Husin F, Mose JC, Setyono IL, Relationship betweenβHcg hormone levels, anxiety and stress with severe degrees of emesis gravidarum. (2022). doi:10.30574/gscbps

13. Mahmood H, Shah TZ, Rasool S, Waqar A, Zia-ul-Miraj A, Maken ZH, et al., Effect of vitamin b6 and acupressure on vomiting symptoms in pregnant women with hyperemesis gravidarum. *American Journal of Biomedical and Life Sciences*, (2021) 9(1) 29. doi: 10.11648/j.ajbls.20210901.14

14. Kabir S, Basher M, Akhter H, Latif T, Akhter S, Karmoker R, et al., Clinico-biochemical profile of women with hyperemesis gravidarum admitted in a tertiary hospital. *Mymensingh Medical Journal: MMJ*, (2017) 26(3) 483-489.

15. Florentin M, Parthymos I, Agouridis AP, Liamis G, Hyperemesis gravidarum: A benign condition of pregnancy or a challenging metabolic disorder?, *European Journal of Case Reports in Internal Medicine*, (2020) 7(12) 001979. doi: 10.12890/2020_001979

16. Joshi V, Strege PR, Farrugia G, Beyder A, Mechanotransduction in gastrointestinal smooth muscle cells: Role of mechanosensitive ion channels. *American Journal of Physiology-Gastrointestinal and Liver Physiology*, (2021). doi: 10.1152/ajpgi.00481.2020

17. Naik S, Talwar D, Acharya S, Kumar S, Shrivastava D, Hyperemesis gravidarum presenting as severe hypokalemic periodic paralysis and type ii respiratory failure: A different form of thyroid storm?, *Cureus*, (2021) 13(11). doi: 10.7759/cureus.19566

18. Pilz S, Theiler-Schwetz V, Pludowski P, Zelzer S, Meinitzer A, Karras SN, et al., Hypercalcemia in pregnancy due to cyp24a1 mutations: Case report and review of the literature. *Nutrients*, (2022) 14(12) 2518. doi: 10.3390/nu14122518

19. Rey E, Jacob CE, Koolian M, Morin F. Hypercalcemia in pregnancy—a multifaceted challenge: case reports and literature review. *Clinical Case Reports* (2016) 4(10):1001–1008. doi:10.1002/ccr3.646

20. Zhong W, Shahbaz O, Teskey G, Beever A, Kachour N, Venketaraman V, et al., Mechanisms of nausea and vomiting: Current knowledge and recent advances in intracellular emetic signaling systems. *International journal of molecular sciences*, (2021) 22(11) 5797. doi: 10.3390/ijms22115797

21. Ashour AM. Efficacy and safety of ondansetron for morning sickness in pregnancy: a systematic review of clinical trials. *Frontiers in Pharmacology* (2023) 14:1291235. doi:10.3389/fphar.2023.1291235
